# LeGO-LOAM-FN: An Improved Simultaneous Localization and Mapping Method Fusing LeGO-LOAM, Faster_GICP and NDT in Complex Orchard Environments

**DOI:** 10.3390/s24020551

**Published:** 2024-01-16

**Authors:** Jiamin Zhang, Sen Chen, Qiyuan Xue, Jie Yang, Guihong Ren, Wuping Zhang, Fuzhong Li

**Affiliations:** School of Software Technology, Shanxi Agricultural University, Jinzhong 030801, China; zjm817107@163.com (J.Z.); czuxxs@163.com (S.C.); xqyuan0107@163.com (Q.X.); yangjie20210228@126.com (J.Y.); endeavorren@163.com (G.R.); zwping@126.com (W.Z.)

**Keywords:** LiDAR, mobile robot, loopback detection, point cloud alignment, SLAM, orchard environment

## Abstract

To solve the problem of cumulative errors when robots build maps in complex orchard environments due to their large scene size, similar features, and unstable motion, this study proposes a loopback registration algorithm based on the fusion of Faster Generalized Iterative Closest Point (Faster_GICP) and Normal Distributions Transform (NDT). First, the algorithm creates a K-Dimensional tree (KD-Tree) structure to eliminate the dynamic obstacle point clouds. Then, the method uses a two-step point filter to reduce the number of feature points of the current frame used for matching and the number of data used for optimization. It also calculates the matching degree of normal distribution probability by meshing the point cloud, and optimizes the precision registration using the Hessian matrix method. In the complex orchard environment with multiple loopback events, the root mean square error and standard deviation of the trajectory of the LeGO-LOAM-FN algorithm are 0.45 m and 0.26 m which are 67% and 73% higher than those of the loopback registration algorithm in the Lightweight and Ground-Optimized LiDAR Odometry and Mapping on Variable Terrain (LeGO-LOAM), respectively. The study proves that this method effectively reduces the influence of the cumulative error, and provides technical support for intelligent operation in the orchard environment.

## 1. Introduction

With the rapid development of science and technology, humanity has unprecedentedly enjoyed the convenience and comfort brought by intelligent technologies, which have greatly changed the ways of production and life, improving the quality of life. Among these, robots, with their high degree of intelligence and strong adaptability, have made significant contributions to social development, finding applications in fields such as industry, agriculture, the military, and healthcare. Robots are a product of multidisciplinary integration, capable of semi-autonomous or fully autonomous operations. Autonomous mobile robots possess multiple functions including environmental perception, dynamic decision making, path planning, and autonomous navigation, allowing them to execute specific tasks in unknown environments [1]. The application of mobile robots in precision agriculture is increasingly widespread, with significant growth in the use of orchard robots. The tasks of orchard robots mainly include acquiring orchard environmental information, pruning and bagging, targeted spraying, thinning flowers and fruits, and fruit harvesting. These tasks demand high precision in mapping and localization during robot movement, requiring accurate perception of the environment for precise positioning. This is achieved through various sensors mounted on the robot to gather information about its pose and surroundings, constructing an environmental map while moving, and continuously correcting its position. However, agricultural scenes represent a typical unstructured environment, characterized by few distinctive features, uneven terrain, and dynamic objects, presenting new challenges in map construction in such scenarios [2].

Simultaneous localization and mapping (SLAM) is a method for orchard robots to localize themselves and build maps of complex environments [3,4]. In outdoor environments, the global navigation satellite system (GNSS) can provide real-time absolute positioning information [5]. However, in orchard scenes with dense canopies, the signal is weak and easily lost, resulting in unreliable positioning. The performance of the Kalman filter is reduced, and the application of the smooth change structure filtering algorithm is limited, which makes the information abnormal. To address the issue of decreased accuracy in conventional Kalman filtering (KF) due to system modeling and noise uncertainty, the Habibi team proposed a model-based robust filtering algorithm Smooth Variable Structure Filter (SVSF). This algorithm employs a discontinuous gain in a variable structure form, ensuring state estimation converges within the true value range. It offers improved robustness against bounded model and noise uncertainties [6]. According to the sensor type [7], SLAM can be classified into visual SLAM [8,9] and laser SLAM [10,11]. Visual SLAM uses visual sensors to obtain environmental information, with the advantages of low cost and simple structure. However, visual SLAM commonly suffers from the heavy computational sensitivity of long-term light changes and has difficulty tracking visual features stably [12,13,14]. Therefore, it is not suitable for environments with obvious light changes such as orchards. On the contrary, laser SLAM technology, characterized by its maturity and robustness, exhibits great resilience to variations in lighting and signal obstruction. Its capabilities are further enhanced by LiDAR, which boasts strong anti-interference features, high resolution, and rapid response times. These attributes make it exceptionally suitable for application in complex orchard environments [15,16,17].

Among them, loopback detection is the key of laser SLAM [18]. It can determine whether the orchard robot passes through the same location by calculating the point cloud similarity between frames. As the scale of the operating scene increases, the content of the historical frame database increases dramatically, and loopback detection will bring a large computational overhead [19]. Especially in complex orchard environments, the lack of loopback detection may lead to the accumulation of LiDAR SLAM position errors over time, which affects the accuracy and effectiveness of SLAM back-end mapping. Wang et al. [20] proposed a global descriptor LiDAR-Iris for LiDAR point clouds, which generates a binary LiDAR-Iris feature map for each point cloud for fast and accurate loopback detection [21]. However, this method takes a long time to search for loopbacks and fails to differentiate between details in orchard environments with multiple similar features, leading to loopback failure. He et al. [22] proposed a global descriptor of multi-viewpoint projection (M2DP) to describe the point clouds and use it for loopback detection, projecting the 3D point cloud to multiple 2D planes and generating density features for the points in each plane [23]. Then, the left and right singular vectors of these features are used as descriptors. This algorithm outperforms the existing global 3D descriptors in both accuracy and efficiency. However, it takes extra computational overhead to calculate a frame of the point cloud descriptor and cannot be applied to the SLAM framework. Chen et al. [24] proposed a convolutional-neural-network-based (CNN-based) method, OverlapNet, for LiDAR loopback detection. It identifies whether a loopback is detected and the change of yaw angle between two frames of data obtained by LiDAR [25]. However, the training process requires a large scale of input data, including depth images, grayscale images, normal maps and semantically segmented images. This extensive data preprocessing step is both cumbersome and impractical for deployment. Moreover, the deep-learning-based image retrieval method employed is a global approach that requires a lot of disparity data for pre-training. Therefore, this method is not suitable for orchard environments where conditions are often highly similar.

LiDAR Odometry and Mapping in Real-time (LOAM) is the most representative real-time 3D laser SLAM algorithm based on feature matching [26,27]. It has a small amount of computation and motion compensation, but there is no loopback detection and back-end graph optimization, and in complex orchard environments, the position error will accumulate over time, leading to low positioning accuracy or even positioning failure. To solve this problem, Shan et al. [28] added a loopback detection module to find the loopback point by combining the Iterative Closest Point (ICP) and Euclidean distance. It introduced lightweight and ground-optimized processing in feature extraction, along with a lightweight, ground-optimized LiDAR odometry and real-time mapping solution. This is exemplified by the Lightweight and Ground-Optimized LiDAR Odometry and Mapping on Variable Terrain (LeGO-LOAM) method, which attains comparable or better accuracy while minimizing computational resource usage. However, its loopback detection algorithm, which relies on ICP matching, faces challenges in terms of time-intensive matching processes and limited real-time performance capabilities. In complex orchard environments, there is a large cumulative error in the initial value of the loop frame obtained by recursion, and the initial value will still fall into the local optimum, making the process hardly converge to the correct result [29,30]. We propose to integrate the Faster_GICP and NDT algorithms on the basis of the LeGO-LOAM algorithm for loopback alignment, aiming to reduce the number of features used for matching and the number of correlations used for optimization for subsequent matching, thereby improving the accuracy and efficiency. We use a chassis to build a software and hardware system, and the performance of the algorithms is verified by field-building experiments under the robot operating system (ROS).

## 2. Algorithm Principle and Improvement

### 2.1. LeGO-LOAM Algorithm

**Figure 1:**
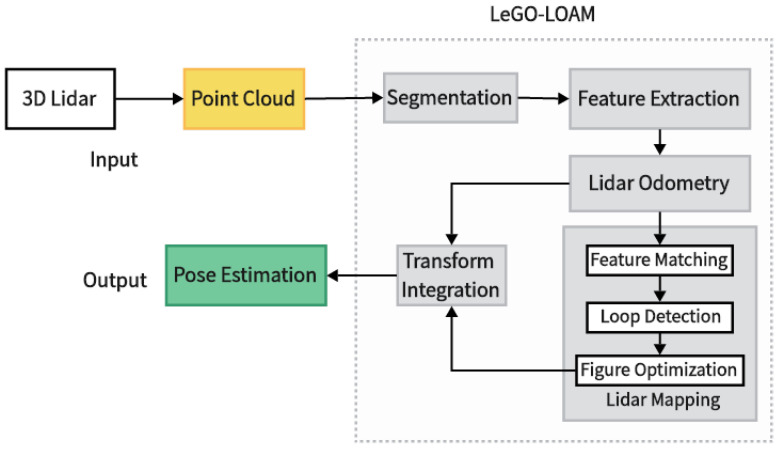
LeGO-LOAM algorithm flow.

LeGO-LOAM is a lightweight real-time positioning and mapping algorithm based on 3D LiDAR, which mainly consists of five parts: point cloud segmentation, feature extraction, LiDAR odometry, LiDAR mapping, and pose integration. It takes 3D LiDAR point clouds as the inputs and outputs 6DoF position estimation [31]. As shown in Figure 1, firstly, the collected orchard environment point cloud is clustered and segmented, and the ground point cloud is separated. At the same time, a small number of point cloud clusters are filtered out. The two-step Levenberg-Marquardt (L-M) optimization method is used to solve the transformation of six degrees of freedom between consecutive frames. The first step uses the ground point cloud to estimate the plane transformation parameters, while the second step matches the edge points and surface points in the segmented point cloud to obtain the pose transformation matrix, further process, and register. Finally, it performs loopback detection to correct the motion estimation drift, and outputs the final pose estimation.

### 2.2. Loopback Detection Algorithm

**Figure 2:**
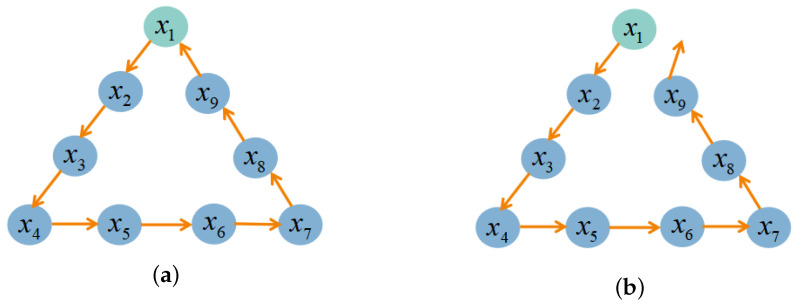
Schematic diagram of loopback detection: (**a**) loopback detection success; and (**b**) loopback detection failure.

In the SLAM approach, the orchard robot position estimation is a recursive process. This means that the current frame position is calculated from the pose of the previous frame. The essence of loopback detection is to find the similarity alignment between two frames of the point cloud [32] in the motion process of the orchard robot, finding where the current point cloud frame $x_{j}\left(j=1,2,\ldots ,N-C\right)$ is similar to the historical frame $x_{i}\left(i=1,2,\ldots ,N\right)$ in real time during the movement of the orchard robot, and construct the position constraints between the loopback pairs, as shown in Figure 2a. To ensure the reliability of loopback detection, the historical frame of the query needs to maintain a certain distance from the current frame *C*. Over time, the positional error will accumulate and lead to trajectory drift [33], as shown in Figure 2b. The loopback detection is very effective in detecting the trajectory drift and can significantly reduce the cumulative errors of map building and localization, making the orchard robot perform obstacle avoidance navigation and other tasks more accurately and quickly. Therefore, it is essential to perform loopback detection for trajectory optimization on large-scale SLAM tasks in orchard environments [34]. The following is an example of how to perform loopback detection for trajectory optimization.

### 2.3. Improved Algorithm Principle

**Figure 3:**
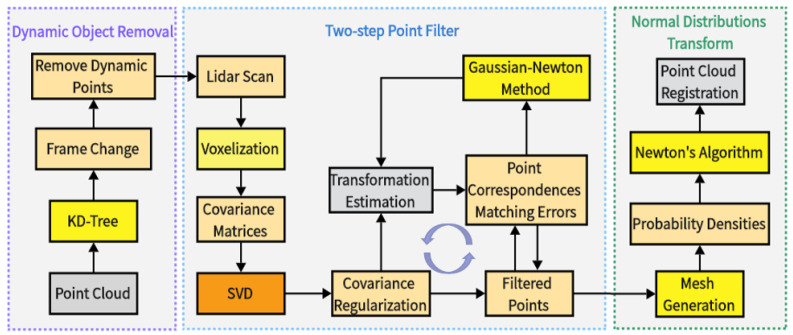
LeGO-LOAM-FN improved process.

The flow of the proposed method in this study is shown in Figure 3. The front-end odometer maintains a sub-map. Before feature extraction, the point cloud uses KD-Tree to check dynamic objects and remove them. Then, loopback detection is performed, and a two-step point filter is implemented using the accept–reject sampling mechanism according to the covariance matrix SVD results [35]. Only the points with high planarity are retained to reduce the distribution approximation error. Then, a further iterative filtering is performed according to the matching error of the LiDAR points in the attitude estimation optimization to remove the point cloud that contributes less to the positional optimization and to improve the alignment efficiency. In order to further reduce the deviation, the NDT alignment is performed on this basis to improve the accuracy of the alignment [36,37], the target point cloud is voxelized, followed by normal distribution fitting based on the points in each voxel grid. The original point cloud is transformed and projected into the grid to calculate the probability density of the point cloud. Subsequently, the Hessian matrix method is used to find the minimum value of the point cloud probability distribution function, and then the optimal transformation matrix is obtained. Finally, it judges whether the termination conditions are satisfied to complete the fine registration of the point cloud.

The LeGO-LOAM-FN algorithm flow is as follows:

(1) The system reads the point cloud data collected by LiDAR, and the points are projected as a depth image. Then, it estimates the ground plane of the depth map, extract the ground points, and the ground points are marked as base points. Each remaining point cloud $K_{t}$ is divided into different clusters by the point cloud segmentation module and marked as segmentation points. Point cloud clusters with a number of point clouds less than 30 were filtered out and different labels were assigned to retained point cloud clusters.
(2) Since dynamic objects are easy to be regarded as feature points, in order to reduce errors, the front-end odometer maintains a sub-map and uses K-Dimensional tree (KD-Tree) to establish a spatial index structure, create two vectors to store the information of the searched proximity points (one to store the index of the point, and one to store the square of the distance of the point), and set the radius search threshold to find the dynamic points to be culled out by the change between two frames.
(3) A two-step point filter based on acceptance–rejection sampling is used to exclude points that rarely benefit LiDAR range performance. Specifically, the LiDAR point cloud is first filtered, retaining only points with high planarity. The process is as follows:
  (a) First, calculate the covariance matrix *C* for each point and perform the singular value decomposition (SVD):
(1) $$\left.C_{i}^{t}=\left[\hat{a},\hat{b},\hat{c}\right]\times \left[\begin{array}{ccc} \lambda_{0} & 0 & 0\\ 0 & \lambda_{1} & 0\\ 0 & 0 & \lambda_{2} \end{array}\right.\right]\times {\left[\hat{a},\hat{b},\hat{c}\right]}^{T},$$
    where *i* is the orchard point cloud serial number: i=1,2,…,n . Use the transformation matrix *T* in the homogeneous coordinate system to describe the “displacements” and “rotations”, and use non-linear optimization (Gauss–Newton method) to find the optimal matching *T*. The eigenvalues are $\lambda_{0},\lambda_{1},\lambda_{2}$ obtained and sorted in descending order, and $\hat{a},\hat{b},\hat{c}$ are the corresponding eigenvector.
  (b) In the first step of filtering, first accept projection sampling of the orchard point cloud. Specifically, the maximum and minimum eigenvalues of $\lambda_{0}$ and $\lambda_{2}$ are normalized to obtain the “roughness” ${\bar{\lambda }}_{2}=\frac{\lambda_{2}}{\lambda_{0}}$ : the smaller the value, the better the planarity, the smaller the roughness, and the smaller the probability of being rejected; on the contrary, the larger the value of ${\bar{\lambda }}_{2}$ , the larger the roughness and the larger the probability of being rejected. Each point is modeled probabilistically whether it is rejected or not. Suppose that the roughness obeys the Gaussian distribution (target distribution):
(2) $$f\left(P\right)=\frac{1}{\sqrt{2\pi }\sigma }\exp\left(-\frac{{\left(\bar{\lambda_{2}}-0\right)}^{2}}{2\sigma^{2}}\right),$$
    where σ is a hyperparameter that needs to be preset. Then define a proposed distribution, where the orchard LiDAR points are directly defined as a uniform distribution:
(3) $$g\left(P\right)=\frac{1}{N},$$
    where $N^{\prime }$ is the number of orchard feature points. In addition, it is also necessary to define a constant $c=\frac{N^{\prime }}{\sqrt{2\pi }\sigma }$ such that the highest point of $c*g\left(P\right)\geq f\left(P\right)$ and thus $f\left(P\right)$ coincides with c∗g(P) , and other values are strictly less than c∗g(P) . If the *i*th orchard feature point $P_{i}^{t}$ is regarded as a sample, then the corresponding $f\left(P_{i}^{t}\right)$ and $c*g\left(P_{i}^{t}\right)$ can be calculated, and $0<\frac{f\left(P_{i}^{t}\right)}{c*g\left(P_{i}^{t}\right)}\leq 1$ can be obtained according to the above inequality, which can be regarded as a probability value $f\left(P_{i}^{t}\right)$ . The larger the value, the closer the sampling is to the target distribution, and it is considered that the *i*th feature point has the probability of $\frac{f\left(P_{i}^{t}\right)}{c*g\left(P_{i}^{t}\right)}$ obeying the distribution $f\left(P\right)$ . Therefore, sampling on the uniform distribution of [0, 1] obtains a probability value μ which can be considered as a probability value if
(4) $$\mu \leq \frac{f\left(P_{i}^{t}\right)}{c*g\left(P_{i}^{t}\right)}.$$
    The *i*th feature point is considered to obey the distribution $f\left(P\right)$ , which is retained. Otherwise, it will not participate in the following optimization.
  (c) Then, optimize the pose estimation, with further iterative filtering based on the contribution of the LiDAR points to the optimized objective function, where the matching error of the point defines its contribution. The key step in the optimization process is to construct iterative updating equations and calculate an association error for each data association result, which is defined as
(5) $$d_{i}=d_{i}^{T}{\left(C_{i}^{t-1}+TC_{i}^{t}T^{T}\right)}^{-1}d_{i},$$
    similar to expression (2) defining the target Gaussian distribution $f\left(d_{i}^{t}\right)$ , and the proposed uniform distribution $g\left(d_{i}^{t}\right)$ and the constant $C_{d}$ , and sampling on the uniform distribution at [0, 1] obtains $\mu_{d}$ . The difference is that when $\mu_{d}\leq \frac{f\left(d_{i}^{t}\right)}{c_{d}*g\left(d_{i}^{t}\right)}$ , the correlation results are rejected because correlation results with small residuals contribute less to the pose optimization and they need to be eliminated. The sampling process reduces the number of points involved in the transform optimization process.
(4) NDT registration is performed to improve accuracy so that the result after fine alignment can meet the preset constraints. The process is as follows.
  The filtered orchard 3D point cloud dataset is divided into a number of fixed-size 3D cubes. Each cube contains at least five point clouds, and the mean *q* and covariance matrix *C* are derived within each cube, respectively:
(6) $$q=\frac{1}{n}\sum_{i=1}^{n}x_{i},$$
(7) $$C=\frac{1}{n-1}\sum_{i=1}^{n}\left(x_{i}-q\right){\left(x_{i}-q\right)}^{T},$$
  where *n* is the number of point clouds in the orchard cube; *i* is the point cloud number i=1,2,…,n ; $x_{i}$ is the point cloud in the matching orchard point cloud cube. The discrete point cloud is represented as a segmented continuously differentiable representation in the form of probability density, and then the probability density (PDF) of each point location in the orchard cube is represented by the NDT algorithm:
(8) $$p\left(x\right)=\frac{1}{m}\exp\left[-\frac{{\left(x-q\right)}^{T}C^{-1}\left(x-q\right)}{2}\right],$$
  where *m* is a constant. Create the probability value NDT of the first frame laser radar scanning point falling into the box, and then use the odometer to initialize. The samples of the second frame are mapped to the first scanning coordinate system according to these coordinate transformation parameters, the probability density of each point is summed and the mathematical expression of the evaluation coordinate transformation parameters is
(9) $$s\left(p\right)=\sum_{i=1}^{n}p\left[T\left(p,x_{i}\right)\right]=\sum_{i}\exp\left[-\frac{{\left(x_{i}-q\right)}^{T}C^{-1}\left(x_{i}-q\right)}{2}\right],$$
  The Hessian matrix method is used to optimize s(p) and then remapped to the loopback detection frame coordinate system until the convergence condition is satisfied. In finding the optimal solution for s(p) , it can be solved by minimizing s(p) and the problem of solving the optimal transformation of the matrix is viewed as the s(p) minimization process. Jump to expression (9) to continue the loopback until the convergence condition is satisfied and the optimal solution is obtained.
(5) The transform integration module combines the results from the LiDAR odometry module and the LiDAR mapping module, and outputs the final position estimation.

## 3. Complex Orchard Environment Test

### 3.1. Test Equipment

**Figure 4:**
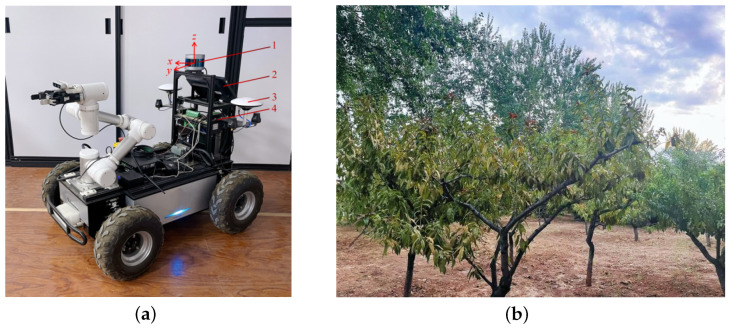
Field orchard environmental experiment: (**a**) orchard robots; and (**b**) orchard environment. 1 3D LiDAR; 2 display; 3 GNSS receiver; and 4 industrial controller.

The experimental platform for map construction is an orchard robot shown in Figure 4a, and the hardware system mainly consists of 3D LiDAR (RS-LiDAR-16), and Jetson AGX Xavier equipped with Ubuntu18.04 operating system. RS-LiDAR-16 is a 16-line digital LiDAR redlaunched by RoboSense in Shenzhen, China. Jetson AGX Xavier is made by NVIDIA in Santa Clara, California, USA. And LiDAR is mounted directly above the mobile chassis, with a horizontal field-of-view (FOV) angle of 360°, a rotational speed of 10–20 Hz, a vertical field of view of ±15°, a vertical angular resolution of 2°, a horizontal angular resolution of 0.1°, an outgoing point count of up to 300,000 points/s, a maximum range of 150 m, and a ranging accuracy of ±2 cm. A body coordinate system *V* is defined to satisfy the right-hand rule. The 3D LiDAR center is taken as the coordinate origin *O*, the x-axis points to the front of the robot, the y-axis is parallel to the wheel axis of the robot and points to the left, and the z-axis points vertically to the upper part of the robot. The orchard point cloud data are recorded, and then the two algorithms are integrated into the robot operating system to evaluate the performance of the two algorithms, LeGO-LOAM and FN-LeGO-LOAM, in different scenarios in terms of the position estimation error.

### 3.2. Test Environment

The data used for the experiments in this paper are the KITTI dataset in autonomous driving [38] and data collected through the field. As shown in Figure 4b, taking the peach orchard of Shanxi Agricultural University as the test site, the environment was scanned, and the orchard robot was manually remote-controlled to travel through the orchard at a speed of 0.5–1 m/s, and the 3D LiDAR recorded the point cloud data at a frequency of 10 Hz. The KITTI dataset has become a benchmark dataset to verify the performance of vision and laser SLAM algorithms outdoors.

The test dataset adopts the sequence of urban road 00, with a scale of 4541-bit poses; Scene 1, with a scale of 1870-bit poses; Scene 2, with a scale of 4604 robots poses, where the terrain in the scene is flat, and the orchard robot moves smoothly without a large amount of jitter; and Scene 3 with a scale of 2334 robot poses, and the experiment simulates the frequent steering and jittering of the orchard robot in the actual picking task.

## 4. Results and Analysis

### 4.1. Test Results

**Figure 5:**
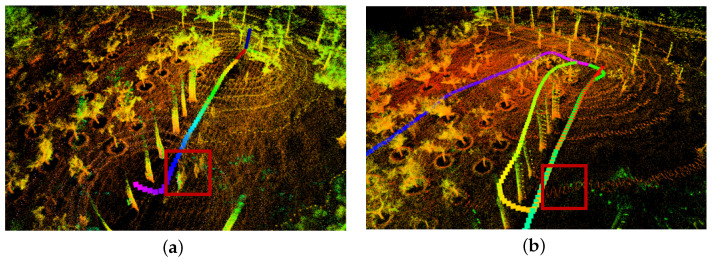
Dynamic object removal: (**a**) LeGO-LOAM algorithm; and (**b**) LeGO-LOAM-FN algorithm.

In the experiment of Scene 3, the point cloud map of the actual environment generated by the robot based on the LeGO-LOAM algorithm is shown in Figure 5a, and the dynamic objects form the double image in the point cloud map. The point cloud map of the actual orchard environment generated by the robot based on the LeGO-LOAM-FN algorithm of this paper is shown in Figure 5b where the dynamic point cloud is eliminated in real time (framed in red), and the processed point cloud is used for map building to reduce the influence of the dynamic objects in the orchard on the map building and to improve the matching accuracy.

**Figure 6:**
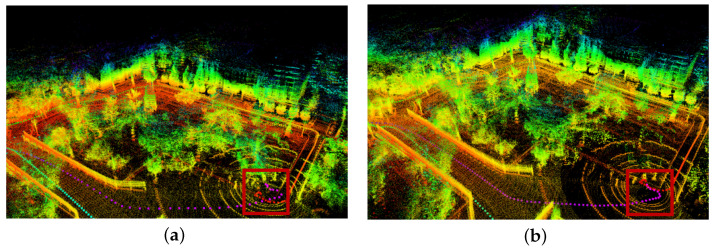
Loopback detection test: (**a**) LeGO-LOAM algorithm; and (**b**) LeGO-LOAM-FN algorithm.

In the loopback detection experiment of Scene 2, the point cloud map of the real environment generated by the robot based on the LeGO-LOAM algorithm is shown in Figure 6a. The loopback identification fails when the orchard robot returns to the starting point, the end point of the radar odometer does not form a closed loopback with the starting point, and the cumulative error of the position estimation leads to the drift of the robot’s trajectory. The point cloud map of the actual orchard environment generated based on the LeGO-LOAM-FN algorithm in this paper is shown in Figure 6b. The robot recognizes the scene it has reached before and successfully performs the positional loopback correction to make the trajectory closed loopback (framed in red).

### 4.2. Test Analysis

In order to evaluate the effectiveness of the proposed LeGO-LOAM-FN mapping method in a field orchard, GNSS continuous acquisition of latitude and longitude information is used as the standard trajectory information to compare with the algorithm output trajectory. In order to visually quantify the algorithm performance, this paper evaluates the two laser SLAM systems in terms of absolute trajectory error (ATE) between the algorithm trajectory and the true value trajectory [39], and takes root mean square error (RMSE) and standard deviation (STD) as the evaluation indices of the algorithms, which are used to measure the deviation of the observed value from the true value, and the smaller value indicates that the map trajectory is closer to the GNSS track.

The RMSE can characterize the building trajectory accuracy with the mathematical expression

(10) $$RMSE=\sqrt{\frac{\sum_{a=1}^{d}{\left(X_{a}-X^{\prime }\right)}^{2}}{d}},$$

where $X^{\prime }$ is the real value of the position of the orchard robot; $X_{a}$ is the measured value; *d* is the number of positions of the orchard robot; *a* is the position serial number; and a=1,2,…,d .

The STD reflects the degree of dispersion of the build trajectory error, and the mathematical expression is

(11) $$STD=\sqrt{\frac{\sum_{a=1}^{d}{\left(X_{a}-\bar{X}\right)}^{2}}{d-1}},$$

where $\bar{X}$ is the mean value.

**Figure 7:**
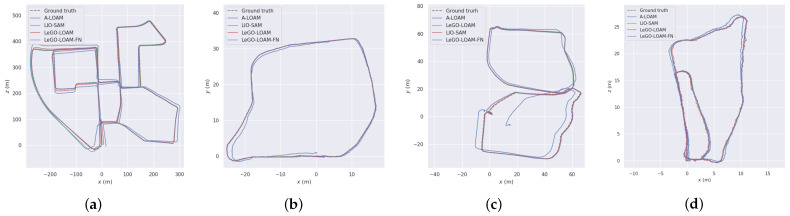
Trial traces versus real-value traces: (**a**) KITTI 00; (**b**) Scene 1; (**c**) Scene 2; and (**d**) Scene 3.

The motion trajectory of the orchard test was estimated by four algorithms: A-LOAM, LIO-SAM, LeGO-LOAM, and LeGO-LOAM-FN. As shown in Figure 7, the algorithm had the best effect and was basically consistent with the true trajectory (ground truth).

Table 1 shows the estimated path length; time consumption; the CPU occupancy rate; RMSE; and STD of A-LOAM, LIO-SAM, LeGO-LOAM, and LeGO-LOAM-FN, reflecting the real-time robustness and stability of the algorithm.

The test found that the map built by LeGO-LOAM-FN algorithm was clearer, the loop effect was better, the estimated trajectory generated was smoother, the overall positioning was more accurate, and the trajectory length was closer to the real trajectory length. Compared with LeGO-LOAM algorithm, the CPU occupancy was reduced by 4% on average, and had better real-time performance, and reduced the time consumption by about 5%. In KITTI 00, the root mean square error and standard deviation of the positioning trajectory of the LeGO-LOAM-FN algorithm are 2.36 m and 0.88 m, respectively, which are reduced by 53% and 57%. In Scene 1, the root mean square error and standard deviation of the proposed algorithm are 0.16 m and 0.08 m, respectively, which are reduced by 24% and 27% compared with the LeGO-LOAM algorithm. In Scene 2, the root mean square error and standard deviation of the LeGO-LOAM-FN algorithm in this paper are 0.25 m and 0.14 m, respectively, which is reduced by 36% and 39% compared with the LeGO-LOAM algorithm trajectory. In Scene 3, the LeGO-LOAM-FN algorithm uses an improved loopback detection algorithm, which is more accurate and efficient compared with the baseline LeGO-LOAM algorithm. The optimization of robot position estimation is obvious. The root mean square error and standard deviation of the position estimation trajectory of the LeGO-LOAM-FN algorithm are 0.45 m and 0.26 m, respectively, which are reduced by 67% and 73%.

In the small-scale scene with a single loopback (Scene 1), the cumulative error of the robot position estimation is small and the optimization space is limited. However, in complex large-scale, multi-loopback scenes (KITTI00, Scene 2, Scene 3), multiple successful loopback registrations by the LeGO-LOAM-FN algorithm also ensure the robot positioning accuracy. In the large-scale agricultural scene, the cumulative error of multiple successful loopback detections and corrections of the orchard robot is significantly reduced, which improves the efficiency of the algorithm. In other words, the algorithm is not limited by the scale of the scene, and the experimental results demonstrate the feasibility of the LeGO-LOAM-FN algorithm for loopback registration in the farmland scene.

## 5. Conclusions

The present study addresses the intricate challenges encountered in simultaneous localization and mapping (SLAM) within the context of complex orchard environments. Specifically, the research focuses on issues arising from long loopback scenes, which often lead to failures in loopback closure. To address these challenges, this study proposes a novel mapping approach based on the LeGO-LOAM-FN algorithm. The effectiveness of the proposed method is rigorously assessed through experiments conducted on both the KITTI dataset and in diverse complex orchard settings. Employing a KD-Tree representation, the algorithm meticulously constructs maps based on odometry, systematically removes dynamic objects, and strategically divides the loop closure detection process for large-scale scenes into two discerning steps. In the initial step, Faster_GICP is employed to selectively eliminate point clouds exhibiting weak planarity and contributing minimally to pose optimization. Subsequently, a refined loopback closure alignment is achieved through the application of small-grid NDT, thereby enhancing the precision of registration. The experimental results affirm the algorithm’s distinct advantages in navigating uneven, large-scale agricultural terrains. The proposed approach effectively mitigates cumulative pose estimation errors, ensuring a harmonious alignment between robot motion trajectories and ground-truth trajectories. Notably, the proposed method consistently outperforms its counterparts in multi-loop scenes, with the root mean square error and standard deviation reduced to 0.45 m and 0.26 m, respectively. This represents a substantial enhancement of 67% and 73% over the baseline LeGO-LOAM algorithm, thereby satisfying the rigorous requirements for point cloud mapping in complex orchard environments.

## Figures and Tables

**Table 1:** Test error comparison between different algorithms.

Scene	Algorithm	Path Length (m)	Time Consumption (s)	The CPU Occupancy Rate (%)	RMSE (m)	STD (m)
Kitti00 (4541)	A-LOAM	3737.584	558.365	69	7.81	4.13
	LIO-SAM	3728.573	563.882	63	3.61	1.91
	LeGO-LOAM	3731.198	570.167	65	5.04	2.04
	LeGO-LOAM-FN	3727.482	541.702	61	2.36	0.88
#1 (1870)	A-LOAM	161.227	195.835	59	0.87	0.54
	LIO-SAM	157.836	196.433	56	0.19	0.10
	LeGO-LOAM	158.209	201.961	557	0.21	0.11
	LeGO-LOAM-FN	155.437	192.382	53	0.16	0.08
#2 (4604)	A-LOAM	440.294	488.274	65	1.62	1.05
	LIO-SAM	449.869	497.239	63	0.45	0.34
	LeGO-LOAM	445.286	489.977	61	0.39	0.23
	LeGO-LOAM-FN	437.761	469.536	56	0.25	0.14
#3 (2334)	A-LOAM	231.802	341.463	67	2.01	1.47
	LIO-SAM	223.293	343.803	63	1.47	1.21
	LeGO-LOAM	226.875	339.368	62	1.36	0.98
	LeGO-LOAM-FN	217.663	321.779	59	0.45	0.26

## Data Availability

The datasets used in the current study are available from the authors on reasonable request. The data are not publicly available due to they are original data and need to be kept confidential.

## Abbreviations

**Acronym**	**Full Name**
LeGO-LOAM-FN	An Improved Simultaneous Localization and Mapping Method Fusing
	LeGO-LOAM, Faster_GICP, and NDT in Complex Orchard Environments
Faster_GICP	Faster Generalized Iterative Closest Point
NDT	Normal Distributions Transform
KD-Tree	K-Dimensional Tree
LeGO-LOAM	Lightweight and Ground-Optimized LiDAR Odometry and Mapping on
	Variable Terrain
SLAM	Simultaneous Localization and Mapping
KF	Kalman Filter
SVSF	Kalman Smooth Variable Structure Filter
GNSS	Global Navigation Satellite System
LOAM	LiDAR Odometry and Mapping in Real Time
ICP	Iterative Closest Point
ROS	Robot Operating System
L-M	Levenberg-Marquardt
A-LOAM	Advanced LiDAR Odometry And Mapping
LIO-SAM	Tightly Coupled LiDAR Inertial Odometry via Smoothing and Mapping
ATE	Absolute Trajectory Error
RMSE	Root Mean Square Error
STD	Standard Deviation
